# Cost-effectiveness of microprocessor-controlled prosthetic knees in the Dutch healthcare system: The impact of study duration on outcomes

**DOI:** 10.33137/cpoj.v9i1.46669

**Published:** 2026-03-05

**Authors:** A Kuhlmann, A Kannenberg

**Affiliations:** 1 Institute of Social Medicine and Epidemiology, University of Lübeck, Germany.; 2 Clinical Research & Services, Ottobock Healthcare LP, Austin, TX, USA.

**Keywords:** Health-Economic Analysis, Health-Utility Analysis, Incremental Cost-Utility Ratio, ICUR, Prosthesis, Prosthetic Knee, Microprocessor-Controlled Prosthetic Knee, MPK, Prosthetics

## Abstract

A previous health-utility analysis of microprocessor-controlled prosthetic knees (MPKs) in the Netherlands used a very short evaluation period of only 6 months resulting in an extremely high incremental cost-utility ratio (ICUR) of €457,063 per quality-adjusted life year (QALY) gained. However, for proper health-economic analyses, an appropriate time horizon, usually the useful life of an intervention, must be chosen to avoid biased results. Therefore, we performed a basic cost-utility analysis (CUA) extrapolating and properly discounting the utility and cost data reported by that article over the useful lifetime of MPKs of 6 years and found an ICUR of €17,945 per QALY. To further refine the CUA, we conducted a modeling study of the ICUR of the MPKs over a timeframe from 6 months to 25 years using existing and published population models for Germany and Sweden. In the modeling study, the ICUR was higher for the Netherlands with €37,567 per QALY than for Germany (€18,276 per QALY) or Sweden (€11,147 per QALY) but below the Dutch threshold for willingness to pay of €50,000 per QALY for people with a lower-limb amputation. Thus, the MPKs were cost-effective for the Dutch healthcare system in all scenarios of the cost-utility analysis. Our analysis showed that time had a much bigger impact on the ICUR than utility with the MPKs becoming cost-effective with the ICUR falling below the €50,000-per-QALY threshold after 4-5 years of use. Thus, a proper time horizon covering the useful lifetime of an intervention is key for health-economic analyses to prevent potentially misleading results and unjustified detrimental decisions for patients.

## INTRODUCTION

In times of aging populations and rapid advancements in medical technologies, health-economic analyses are crucial for evaluating the value for money of healthcare interventions and supporting optimal resource allocation. A recent study of Bosman et al.,^1^ published in the Canadian Prosthetic and Orthotic Journal, and their response to a letter to the editor criticizing its methodology^2,3^ has questioned the health-economic value of microprocessor-controlled prosthetic knees (MPK) for individuals with unilateral above-knee amputations under the conditions of the healthcare system in the Netherlands. Though the effort and most of the methodology is commendable, the authors chose to select an extremely short evaluation period of only 6 months, while the useful lifetime and replacement cycle of MPKs in the Netherlands is 6 years. As the useful lifetime of an intervention or device is a crucial parameter that interacts directly with the evaluation process, the longevity of a medical device or the durability of an intervention's effect determines the time horizon over which costs and benefits accrue.

According to good practice recommendations^4^ and the new national Dutch guideline for health-economic evaluations,^5^ the time horizon must be long enough to capture the difference between all costs and effects associated with the intervention. Hence, for interventions with long-lasting effects, a longer time horizon is necessary to capture all relevant costs, benefits, and consequences. Ultimately, accurately defining the useful lifetime and applying suitable discount rates for future benefits and cost along with sensitivity analyses to test the robustness of results against different rates is essential for a reliable health-economic evaluation. These methods ensure that decision-makers have a comprehensive understanding of the long-term value and resource implications of an intervention or device, balancing immediate investments against future health gains and cost or savings.^6–9^

A critical and often complex aspect of these evaluations is the necessity to extrapolate short-term clinical trial data, typically gathered over 6 or 12 months, to longer time horizons. This is necessary because the long-term impact of a treatment on costs, survival, and quality of life can often not be fully observed within the limited duration of a trial.^10,11^

Bosman et al.^1^ chose a 6-month evaluation period to avoid a recall bias for data collection beyond 6 months in the past and did not extrapolate the data into the future. Criticized for this methodology,^2^ they argue that any “proposed corrections to our ICUR [incremental cost-utility ratio] are based on assumptions beyond our dataset; they illustrate the sensitivity of ICUR to prosthesis life-cycle assumptions”.^3^ However, evaluating the health-economic value of any advanced medical intervention over such short period of time would probably result in most non-life-saving interventions being considered not cost-effective. For example, a study evaluated the health-economic value of a total knee replacement (TKA) over an average timeframe of 11 years (deducted from the data reported) in a Medicare population with an average age of 74 years (range 68-70 years) and found an incremental cost-utility ratio (ICUR) of the TKA of US$18,300 per QALY.^12^ However, shortening the evaluation window to 1/12 of that reasonable timeframe as done by Bosman et al.^1^ would have inflated the ICUR to US$155,756 per QALY in 11 months. Contrary to the claim of Bosman et al. that assumptions were beyond their dataset,^3^ extrapolation of utility and cost data into the future is an established and accepted method of health-economic analyses,^8,9^ In fact, we are not aware of any health-economic analysis that collected utility and cost data over the entire evaluation period. Nevertheless, Bosman et al.^1^ could have inaugurated an innovative way of approaching health-economic analyses by answering the legitimate research question: After what duration of use are MPKs cost-effective, if any? However, this question was not addressed in their analysis. Therefore, this professional opinion article attempts to answer this question using their reported data.

## METHODOLOGY

### Basic Cost-Utility Analysis

Based on the data of Bosman et al.,^1^ we performed a “basic” cost-utility analysis of the MPKs with extrapolation and discounting over a period of 6 years, the typical useful lifetime and replacement cycle of MPKs in the Netherlands. In this basic analysis, we did not consider mortality or sensitivity of data variation as factors. The base data was extrapolated from 6 months to a full year for year 1 and then for years 2 to 6 with two scenarios:

- In **scenario 1**, we chose the originally reported utility difference of 0.045 in favor of the MPKs as the basis. We multiplied the utility difference of 0.045 with 1 year resulting in 0.045 QALY gained by the MPKs in year 1.
- In **scenario 2** we chose the reported 6-month QALY difference after bootstrapping of 0.032 QALY in favor of the MPKs as the basis. We multiplied the 6-month QALY difference of 0.032 QALY by 2 resulting in 0.064 QALY gained by MPK use in year 1.

For years 2-6, the utility/QALY gained in year 1 was discounted by 1.5% per year using the official Dutch guideline.^5^ Discounting is a core principle in health-economic analyses as it makes future costs, benefits, and health outcomes comparable to those occurring in the present. This process acknowledges the “time value of money” and reflects that people generally prefer benefits sooner rather than later (time preference). More fundamentally, discounting accounts for the opportunity cost of capital: resources invested in a public health program could otherwise be invested in the market or other sectors, where they would accrue interest and yield a return over time.^6–9^ Costs were determined the same way for both scenarios. For year 1, the 6-month total cost including the prosthesis and the 6-month total cost excluding the prosthesis were added. For years 2-6, the 6-month total cost excluding the prosthesis was doubled. According to the official Dutch Guideline,^5^ future costs were discounted by 3% per year.

In addition to the full 6-year cost-utility analysis, we also performed an annual sensitivity analysis for both scenarios by calculating the ICUR for each year of the useful lifetime of an MPK of 6 years.

### Comparative Modelling Analysis

For the comparative modelling analysis, we used existing and published German and Swedish health economic (HE) models^13,14^ as well as the basic calculation approach to investigate the impact of the time horizon and differences in input data on the results of the evaluation.

**1. Germany:** We used a German HE model and reported base-case parameter values to estimate the cost-utility of the C-Leg vs non-microprocessor-controlled prosthetic knees (NMPKs) in prosthesis users with diabetes mellitus (DM) and users without DM.^13^

**2. Sweden:** We used a Swedish HE model and reported base-case parameter values to estimate the cost-utility of the KENEVO vs NMPKs in prosthesis users (all etiologies).^14^

**3. Netherlands:** We performed cost-utility analyses by extrapolating data extracted from Bosman et al.^1^ (basic calculation approach).

In addition, we informed the German model with the extracted data to perform a cost-utility analysis in prosthesis users with DM. Health outcomes (QALYs) were discounted by 1.5% and costs by 3% per year.^5^ The main differences between the basic calculation approach and the modelling approach was that the basic approach was limited to the useful lifetime of an MPK in the Netherlands of 6 years and did not include user mortality, while the modelling approach included patient survival and different life cycles of the prosthesis types. For the modelling analysis, we used the (German) age distribution and survival times reported in Kuhlmann et al.^13^ and excluded the effects of falling, since these may have already been (partially) included in the cost and QALY estimates of Bosman et al.^1^

**4.** We varied the model period from 0.5 to 25 years to analyze the impact of the time horizon of the evaluation on the HE results.

The data extracted from Bosman et al.^1^ is shown in **Table 1** and the key parameter values and assumptions of the models in **Table 2**.

**Table 1:** Data extracted from Bosman et al., 2025.^1^

	NMPK group	MPK group	Difference
EQ-5D-5L utility	0.742 (n#=49)	0.787 (n=60)	0.045
QALYs in 6 months	0.37 (n=46)	0.40 (n=55)	0.032
6-month cost excluding prosthesis*	€4,981 (n=42)	€3,909 (n=55)	−€1,072
6-month cost including prosthesis*	€9,395 (n=42)	€24,927 (n=55)	€15,532
One-time prosthesis cost	€4,417 (n=42)	€21,018** (n=55)	€16,601
6-month Incremental cost-utility ratio (ICUR)			€457,063 per QALY
Applicable willingness-to-pay threshold for the Netherlands			€50,000 per QALY***

* In the utility analysis (results section), Bosman et al. reported a mean cost difference of €14,626 after boot strapping. Assuming that the prosthesis costs were kept constant during the bootstrap, the difference in the 6-month costs excluding prosthesis costs would even be higher in favor of the MPK (−€1,978). For our analysis, we still used the lower value of (−€1,072).

** According to Livit Orthopedie, one of the two prosthetic companies contributing patient recruitment and cost data to the study of Bosman et al.^1^ and a member of the Ottobock Patient Care network, this cost includes the standard 6-year warranty package for MPKs in the Netherlands.

*** Per the supplements of Bosman et al.,^1^ this threshold would apply to prosthesis users with vascular disease.

# n: number of participants.

**Table 2:** **Key parameters for the models.** Data was extracted from the following studies: Germany: Kuhlmann et al. 2020;^13^ Sweden: Kuhlmann et al. 2022;^14^ Netherlands: Bosman et al. 2025.^1^ Other reported sources refer to the original data which were used in German and Swedish study.

Parameter			Parameter values				Source / comment
**Country**			**Germany**		**Sweden**	**Netherlands**	
**Health economic model**			German	German	Swedish	German	
**MPKs**			C-Leg	C-Leg	Kenevo	not reported	Germany: Kuhlmann et al. 2020.^13^ Sweden: Kuhlmann et al. 2022.^14^ Netherlands: Bosman et al. 2025.^1^
**Etiology**			Without DM*	With DM	all	With DM	
**Population structure**	40-49y		6.3%	5.8%	**-**	5.8%	Germany: Kuhlmann et al. 2020.^13^ Sweden: Kuhlmann et al. 2022.^14^ Netherlands: The German population structure in prosthesis user with DM was used for the modelling approach.
	50-59y		18.7%	17.7%	-	17.7%	
	60-69y (65-74y)		32.9%	31.9%	32.8%	31.9%	
	70-79y (75-84y)		24.0%	24.4%	39.8%	24.4%	
	80+y (85+y)		18.1%	20.2%	27.4%	20.2%	
**Average life expectancy (in years)**			40-49y: 28.0	40-49y: 13.6	-	40-49y: 13.6	Germany: Kuhlmann et al. 2020.^13^ Sweden: Kuhlmann et al. 2022.^14^ Netherlands: German survival data was applied in the modelling approach.
			50-59y: 20.0	50-59y: 9.1	-	50-59y: 9.1	
			60-69y: 13.1	60-69y: 6.0	65-74y: 4.3	60-69y: 6.0	
			70-79y: 7.8	70-79y: 3.6	75-84y: 3.3	70-79y: 3.6	
			80+y: 4.5	80+y: 2.1	85+y: 2.2	80+y: 2.1	
**Utilities**	Annual utility gain of MPK compared with NMPK	<55y	0.119		-	0.064	Germany: Cutti et al. 2017.^19^ Sweden: Cutti et al. 2017.^19^ Netherlands: Bosman et al. 2025.^1^
		55-64y	0.082		-		
		65+y	0.078		0.078		
	Additional disutility for fall-related medical events		Hospitalization, outpatient treatment, fatal falls		Hospitalization, outpatient treatment, fatal falls	Not included	Germany: Kuhlmann et al. 2020.^13^ Sweden: Kuhlmann et al. 2022.^14^ Netherlands: Fall-related medical events were not modelled.
**Prosthesis costs**	Price (Euro)	NMPKs	10,862		1,268	4,417	Germany: Kuhlmann et al. 2020.^13^ Sweden: Kuhlmann et al. 2022,^14^ average life cycle of the NMPKs was obtained from Brodtkorb et al. 2007.^21^ Netherlands: Bosman et al. 2025.^1^
		MPKs	29,645^1^		8,937+4,283^2^	21,018^1^	
	Average life cycle (years)	NMPKs	3.5		2	3.5	
		MPKs	6		3+3	6	
**Medical and non-medical costs**	Cost perspective		Payer		Payer	Societal	
	Annual cost savings of MPKs compared with NMPKs (Euro)		Difference in costs of fall-related medical events		Difference in costs of fall-related medical events	2,144	Germany, Sweden: Calculated by the model Netherlands: Bosman et al. 2025.^1^
**Discounting**	Health outcomes		3%		3%	1.5%	Germany: IQWiG 2025.^15^ Sweden: TLV.^16^ Netherlands: Geuzinge et al. 2025.^5^
	Costs		3%		3%	3%	
	Price year		2019		2019	2023	Germany: Kuhlmann et al. 2020.^13^ Sweden: Kuhlmann et al. 2022.^14^ Netherlands: Bosman et al. 2025; the exact price year wasn't reported.^1^ The study was mainly conducted in 2023.

1 MPK price including six-year warranty,

2 MPK price including three-year warranty + three-year add-on warranty (only if the prosthesis user is still alive)

* Diabetes Mellitus (DM)

## RESULTS

### Basic Cost-Utility Analysis

The results of the basic 6-year cost-utility analyses for scenarios 1 and 2 are depicted in **Table 3** and **Table 4**. The analyses show that in both scenarios, the ICUR for MPKs is way below the applicable Dutch willingness-to-pay threshold of €50,000 per QALY.

**Table 3:** Scenario 1 - 6-year cost-utility analysis of MPKs vs. NMPKs based on a utility difference of 0.045.

Year	Difference in QALY	Difference in cost	Comments
Year 1	0.045	€14,460	Difference in utility X 1 year; Difference in 6-month cost including prosthesis (€15,532) + Difference in 6-month cost excluding prosthesis (−€1,072).
Year 2	0.0443	−€2,080	Difference in utility in year 1 discounted by 1.5%; 2 X 6-month cost excluding prosthesis in year 1 (−€2,144) discounted by 3%.
Year 3	0.0437	−€2,017	Difference in utility in year 2 discounted by 1.5%; 2 X 6-month cost excluding prosthesis in year 2 (−€2,080) discounted by 3%.
Year 4	0.0430	−€1,957	Difference in utility in year 3 discounted by 1.5%; 2 X 6-month cost excluding prosthesis in year 3 (−€2,017) discounted by 3%.
Year 5	0.0424	−€1,898	Difference in utility in year 4 discounted by 1.5%; 2 X 6-month cost excluding prosthesis in year 4 (−€1,957) discounted by 3%.
Year 6	0.0417	−€1,841	Difference in utility in year 5 discounted by 1.5%; 2 X 6-month cost excluding prosthesis in year 5 (−€1,898) discounted by 3%.
**Total**	**0.260**	**€4,667**	**Incremental cost-utility ratio (ICUR) = €17,945 per QALY**

**Table 4:** Scenario 2 - 6-year cost-utility analysis of MPKs vs. NMPKs based on a 6-month QALY difference of 0.032 QALY.

Year	Difference in QALY	Difference in cost	Comments
6-month	0.032		
Year 1	0.064	€14,460	Difference in utility for 6 months X 2; Difference in 6-month cost including prosthesis (€15,532) + Difference in 6-month cost excluding prosthesis (−€1,072).
Year 2	0.063	−€2,080	Difference in utility in year 1 discounted by 1.5%; 2 X 6-month cost excluding prosthesis in year 1 (- €2,144) discounted by 3%.
Year 3	0.062	−€2,017	Difference in utility in year 2 discounted by 1.5%; 2 X 6-month cost excluding prosthesis in year 2 (- €2,080) discounted by 3%.
Year 4	0.061	−€1,957	Difference in utility in year 3 discounted by 1.5%; 2 X 6-month cost excluding prosthesis in year 3 (- €2,017) discounted by 3%.
Year 5	0.060	−€1,898	Difference in utility in year 4 discounted by 1.5%; 2 X 6-month cost excluding prosthesis in year 4 (- €1,957) discounted by 3%.
Year 6	0.059	−€1,841	Difference in utility in year 5 discounted by 1.5%; 2 X 6-month cost excluding prosthesis in year 5 (- €1,898) discounted by 3%.
**Total**	**0.370**	**€4,667**	**Incremental cost-utility ratio (ICUR) = €12,618 per QALY**

The basic annual sensitivity analyses are depicted in **Table 5** and **Table 6**. Applying the threshold of €50,000 per QALY, the MPK starts being cost-effective in year 4 in both scenarios.

**Table 5:** Annual sensitivity analysis for scenario 1.

Time frame	Accrued utility (QALYs)	Accrued cost (€)	ICUR (€ per QALY)
Year 1	0.045	14,460	321,333
Year 2	0.089	12,380	138,599
Year 3	0.133	10,363	77,926
Year 4	0.176	8,406	47,765
Year 5	0.218	6,508	29,806
Year 6	0.260	4,667	17,945

**Table 6:** Annual sensitivity analysis for scenario 2.

Time frame	Accrued utility (QALYs)	Accrued cost (€)	ICUR (€ per QALY)
6 months	0.032	14,626	457,063
Year 1	0.064	14,460	225,938
Year 2	0.127	12,380	97,452
Year 3	0.189	10,363	54,792
Year 4	0.250	8,406	33,585
Year 5	0.310	6,508	20,957
Year 6	0.369	4,667	12,618

### Comparative Modelling Analysis

#### Impact of the time horizon on the results of the evaluation

**Figure 1:**
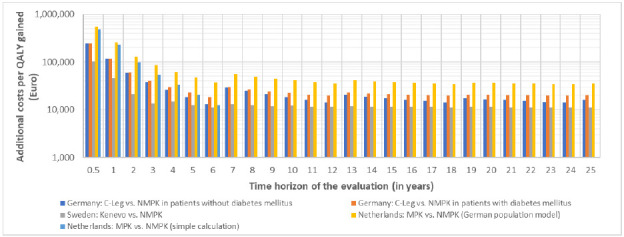
Results for the ICUR modeling scenarios Germany (patients w/ and w/o diabetes mellitus), Sweden, Netherlands (simple calculation) and Netherlands (German population modeling). Please note that the axis “Additional costs per QALY gained” has a logarithmic scale.

The time horizon of the analysis had a substantial impact on the ICUR, QALYs gained and additional costs in all five modeling scenarios. Over the assumed life cycle of the MPKs, the ICUR decreased drastically from a range between €102,532 (Sweden) to €553,552 (Netherlands) per QALY gained after 6 months to a range from €11,147 (Sweden) to €37,567 (Netherlands) after 6 years (**Figure 1**). After seven years, the ICURs increased again in all scenarios since a new life cycle of the costlier MPKs began. Depending on the scenario, the ICURs after seven years were between estimated ICURs after three to five years. From year seven to year twelve, the ICURs decreased again in all scenarios. This pattern could be observed with each life cycle of the MPKs (**Figure 1**). Depending on the scenario, the ICUR estimates began to stabilize after the second or third life cycle of the MPKs for two reasons: 1. The incremental costs and benefits of another year became relatively small compared to those already accumulated, and 2. Many prosthesis users had already died which further decreases the added benefits and costs of another year.

In the simple calculation approach, the ICUR was only computed for time horizons of 0.5-6 years. Since mortality wasn’t considered in this approach, results for longer time horizons would have become extremely unrealistic.

Assuming an ICUR threshold of €40,000 per QALY in Sweden,^17^ the KENEVO became cost-effective after an evaluation period of two years in the Swedish setting. Assuming an ICUR threshold of €50,000 per QALY gained in the Netherlands (supplemental material), the MPKs became cost-effective after five years (German model informed by data from Bosman et al.^1^)

#### Scenario comparison

The comparative modelling results showed substantial variation in ICURs across countries, with the highest ICURs observed in the Netherlands and the lowest in Sweden (**Figure 1, Table S1**). In the Netherlands, the higher ICUR was mainly driven by lower QALY gains (**Table S2**) compared to other scenarios. These lower QALY gains more than offset the substantially higher savings in medical and non-medical costs (**Table S3**). In Sweden, the lower ICUR was primarily explained by a smaller difference in prosthesis costs (**Table S4**). Despite the shorter assumed life cycle of NMPKs (3.5 vs 6 years for the MPKs) in the Dutch modelling approach, the ICUR is higher than in the basic calculation approach due to the inclusion of mortality. Discount rates had a minor impact on ICURs (**Table S1**). However, despite differences in assumptions, MPKs types, study populations etc., ICURs were well below €50,000 per QALY in all scenarios when applying a time horizon of at least six years and showed the same trend to reduce the impact of an additional year of MPKs use over time.

The lowest QALY gains were observed in the Netherlands and the highest in Germany (**Table S1**). The reduced QALY gains observed in the Netherlands were driven by a smaller difference in utilities between MPKs and NMPKs users and by the exclusion of health effects related to falling. In Sweden, utility values for prosthesis use and fall-related outcomes were similar as those used in the German setting, but overall QALY gains were reduced due to higher average mortality in the population (only 65+years were included in the Swedish study).

Non-prosthesis cost savings were highest in the Dutch setting (**Table S3**). This was mainly because the Dutch scenario adopted a societal perspective, capturing a broader range of medical and non-medical costs, whereas other scenarios only included fall-related costs from a payer’s perspective. In addition, in the German and Swedish scenarios a reduction of falls was assumed over time, reducing the annual difference in fall costs by over 50% from year 4 onwards compared with the first year after prosthesis fitting.

## DISCUSSION

Our comparative modelling analysis showed that the time horizon had a huge impact on the results of the cost-utility analysis in all scenarios. Extending the study from 0.5 to 6 or 25 years reduced the ICUR by 89% in the Swedish setting, by 92-95% in the German setting, and 93-94% in the Dutch setting. Despite differences in assumptions, MPKs types, study populations etc., ICURs were well below €50,000 per QALY in all scenarios when applying a time horizon of at least four to five years and showed the same trend to reduce the impact of an additional year of MPKs use over time.

Hence, cost-utility analysis for MPKs over their useful lifetime and replacement cycle in the Netherlands showed that MPKs are very likely cost-effective at the Dutch threshold of €50,000 per QALY as defined by the Zorginstituut Nederland (National Health Care Institute). Our findings are in line with earlier health-economic studies for Italy,^18–20^ Sweden,^14,21^ Germany,^13^ and the United States.^22,23^ The analysis of the impact of the time horizon showed that the time horizon of the study of Bosman et al., 2025,^1^ was way too short to capture the medical and cost benefits of MPK over the useful lifetime and regular replacement cycle in the Netherlands comprehensively. Our findings are especially remarkable because the differences in utility between MPK and NMPK found by Bosman et al. were lower than in previous studies,^13,14,18–24^ resulting in the accrual of lower total utility over time. However, our sensitivity analyses showed that the impact of the time horizon of the evaluation is much greater than that of utility.

As the author group is known for their expertise in providing medical care to patients with amputations and acknowledges the results of previous health-economic studies over longer periods, we find it surprising that they claimed “that assumptions about replacement cycles warrant investigation” and insisted on “very small” differences in utility as the main reason for their astronomical ICUR for 6 months.^3^ Nevertheless, Bosman et al.^1^ found lower total societal costs (excluding prosthesis costs) for the MPK group, which resulted in a reduction in the cost difference between the MPK and NMPK group over time. Though our analysis has limitations, it should be appreciated that we took a very conservative approach to account for the uncertainties of extrapolating present costs and utility values into the future that disadvantaged the MPKs. The model accepted the prosthetic cost assumption extracted from Bosman et al.^1^ and did not include fall-related reductions in hospitalization costs nor fall-related depreciation of QALYs in the NMPK group. Therefore, we are very confident that our finding that MPKs are cost-effective under the conditions of the healthcare system of the Netherlands is reliable.

### Limitations

Our analysis has limitations. As we did not have access to the raw data of Bosman et al.,^1^ we could only work with aggregate data reported in the publication. Thus, sensitivity analyses, such as bootstrapping as performed by the authors of the original publication, could not be conducted. To account for this limitation, we took a very conservative approach to the analysis that disadvantaged the MPKs over time. First, we did not account for the typical replacement of an NMPKs after 3-4 years that would have added costs to the NMPKs side and, thus, further reduced the cost difference over the 6-year time horizon. Second, the costs in the MPK group showed higher costs than in the NMPK group for physical therapy, psychologist/psychiatrist, out-of-pocket cost, and presenteeism in the first 6 months. It is unlikely that these higher costs would persist over the full useful lifetime of an MPK of 6 years. However, we did not adjust these costs in the extrapolation as this would have increased the cost difference excluding the prosthesis cost in favor of the MPKs and resulted in a faster and bigger reduction of the total cost difference over time.

In the comparative modelling analysis, we used German population and mortality data for the Dutch setting. This resulted in a higher average age than in the study by Bosman et al. Increased age is associated with higher ICURs^13,14^ due to higher overall mortality. In addition, as a conservative approach, we applied mortality rates of German amputees with DM which are higher than mortality rates in amputees without DM.

## CONCLUSION

The cost-utility analyses demonstrated that the MPK is likely to improve prosthesis-specific quality of life and utility, but at higher costs. However, the improvement in health-related quality of life, utility, and cost savings accumulate over time. Hence, choosing extremely short time horizons for health economic evaluations of prostheses does not show the real value for money of MPKs. Furthermore, it does neither follow good practice guidelines nor the national Dutch guideline for conducting cost-effectiveness analyses. Using an appropriate time horizon, the ICURs were well below the official willingness-to-pay threshold of €50,000 per QALY gained in all scenarios after four to five years of MPKs use.

## Figures and Tables

**Figure:**
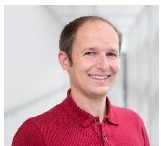
Picture: Universitätsmedizin Halle

**Figure:**
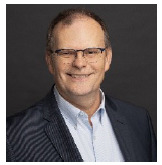


**Table S1:** Incremental cost-utility ratio (additional costs in Euro per QALY gained) of MPKs vs NMPKs.

Time horizon (years)	Germany: C-Leg vs. NMPKs						Sweden: Kenevo vs. NMPKs			Netherlands: MPKs vs. NMPKs		
	With DM			Without DM								
	Discount rates: health outcomes/costs			Discount rates: health outcomes/costs			Discount rates: health outcomes/costs			Discount rates: health outcomes/costs		
0%/0%	1.5%/3%	**3%/3%**	0%/0%	1.5%/3%	**3%/3%**	0%/0%	1.5%/3%	**3%/3%**	0%/0%	**1.5%/3%**	3%/3%
0.5	246,350	246,350	**246,350**	246,128	246,128	**246,128**	102,533	102,533	**102,533**	553,552	**553,552**	553,552
1	117,562	117,562	**117,562**	117,469	117,469	**117,469**	45,151	45,151	**45,151**	260,026	**260,026**	260,026
2	59,592	60,129	**60,526**	58,329	58,867	**59,265**	20,885	21,150	**21,286**	129,872	**131,179**	132,019
3	39,185	40,029	**40,540**	36,726	37,572	**38,076**	13,033	13,440	**13,603**	83,896	**85,965**	87,032
4	28,081	29,215	**29,754**	24,645	25,785	**26,296**	14,534	14,686	**14,938**	59,163	**61,902**	63,019
5	20,814	22,224	**22,758**	16,523	17,946	**18,420**	12,090	12,358	**12,627**	43,771	**47,108**	48,207
6	16,133	17,755	**18,276**	11,208	12,843	**13,268**	10,507	10,866	**11,147**	33,735	**37,567**	38,629
7	29,122	28,765	**29,757**	29,016	28,065	**29,181**	12,947	12,898	**13,280**	55,413	**55,832**	57,668
8	25,636	25,638	**26,648**	24,055	23,640	**24,739**	12,023	12,065	**12,463**	48,439	**49,583**	51,428
9	22,901	23,210	**24,234**	20,104	20,140	**21,212**	11,346	11,462	**11,874**	43,012	**44,789**	46,637
10	20,724	21,298	**22,333**	16,915	17,334	**18,373**	11,554	11,585	**12,033**	38,708	**41,041**	42,889
11	18,984	19,784	**20,830**	14,334	15,075	**16,081**	11,149	11,236	**11,696**	35,254	**38,078**	39,926
12	17,575	18,570	**19,628**	12,219	13,234	**14,205**	10,835	10,967	**11,440**	32,443	**35,702**	37,552
13	21,800	21,603	**22,916**	20,260	19,167	**20,703**	11,299	11,282	**11,789**	39,744	**40,915**	43,159
14	20,668	20,680	**22,012**	18,152	17,463	**18,976**	11,093	11,112	**11,630**	37,592	**39,194**	41,453
15	19,719	19,916	**21,266**	16,350	16,013	**17,506**	10,927	10,977	**11,504**	35,794	**37,780**	40,053
16	18,921	19,280	**20,648**	14,800	14,774	**16,247**	10,968	10,988	**11,530**	34,283	**36,612**	38,900
17	18,245	18,748	**20,133**	13,458	13,707	**15,161**	10,856	10,899	**11,450**	33,007	**35,643**	37,946
18	17,669	18,301	**19,703**	12,289	12,783	**14,219**	10,764	10,828	**11,385**	31,924	**34,836**	37,154
19	19,358	19,314	**20,843**	16,800	15,644	**17,498**	10,888	10,894	**11,464**	34,853	**36,591**	39,089
20	18,904	18,977	**20,523**	15,671	14,811	**16,656**	10,824	10,845	**11,421**	34,027	**36,005**	38,521
21	18,511	18,689	**20,253**	14,671	14,076	**15,914**	10,769	10,804	**11,386**	33,319	**35,512**	38,044
22	18,172	18,444	**20,024**	13,782	13,427	**15,258**	10,779	10,802	**11,391**	32,710	**35,097**	37,644
23	17,878	18,235	**19,830**	12,988	12,851	**14,677**	10,739	10,774	**11,367**	32,186	**34,747**	37,308
24	17,624	18,056	**19,665**	12,278	12,338	**14,161**	10,706	10,750	**11,347**	31,737	**34,452**	37,027
25	18,347	18,413	**20,332**	15,027	13,828	**16,123**	10,747	10,766	**11,369**	32,971	**35,067**	37,721

**In bolt:** recommended country-specific discount rates for the base case analysis.^1,13,14^

**Table S2:** QALYs gained (undiscounted) by MPKs vs NMPKs.

Time horizon (years)	Germany: C-Leg vs. NMPKs		Sweden: Kenevo vs. NMPKs	Netherlands: MPKs vs. NMPKs
	With DM	Without DM		
0.5	0.07	0.07	0.07	0.03
1	0.15	0.15	0.13	0.06
2	0.27	0.27	0.24	0.10
3	0.37	0.39	0.32	0.14
4	0.45	0.49	0.38	0.17
5	0.53	0.60	0.43	0.19
6	0.59	0.70	0.47	0.22
7	0.65	0.80	0.50	0.24
8	0.71	0.90	0.53	0.25
9	0.76	0.99	0.55	0.27
10	0.80	1.09	0.57	0.28
11	0.84	1.19	0.58	0.29
12	0.88	1.28	0.59	0.30
13	0.91	1.38	0.60	0.31
14	0.94	1.47	0.61	0.32
15	0.96	1.56	0.62	0.32
16	0.99	1.65	0.62	0.33
17	1.01	1.74	0.63	0.33
18	1.03	1.82	0.63	0.34
19	1.04	1.91	0.64	0.34
20	1.06	1.99	0.64	0.34
21	1.07	2.07	0.64	0.35
22	1.09	2.14	0.64	0.35
23	1.10	2.22	0.65	0.35
24	1.11	2.29	0.65	0.35
25	1.12	2.36	0.65	0.36

**Table S3:** Medical and non-medical costs (undiscounted) saved by MPKs vs NMPKs.

Time horizon (years)	Germany: C-Leg vs. NMPKs		Sweden: Kenevo vs. NMPKs	Netherlands: MPKs vs. NMPKs
	With DM	Without DM		
0.5	817	817	817	947
1	1,637	1,634	1,635	1,895
2	2,675	2,715	2,588	3,388
3	3,325	3,463	3,120	4,617
4	3,701	3,940	3,392	5,646
5	4,018	4,386	3,595	6,517
6	4,289	4,804	3,748	7,262
7	4,521	5,198	3,863	7,902
8	4,721	5,571	3,951	8,456
9	4,895	5,924	4,018	8,938
10	5,046	6,261	4,071	9,360
11	5,179	6,582	4,111	9,730
12	5,295	6,887	4,142	10,054
13	5,396	7,178	4,167	10,338
14	5,485	7,453	4,186	10,586
15	5,562	7,715	4,202	10,802
16	5,629	7,962	4,214	10,991
17	5,688	8,197	4,224	11,156
18	5,738	8,418	4,232	11,300
19	5,783	8,627	4,238	11,425
20	5,821	8,824	4,243	11,535
21	5,855	9,011	4,247	11,630
22	5,884	9,186	4,251	11,714
23	5,910	9,351	4,253	11,786
24	5,932	9,507	4,256	11,850
25	5,951	9,653	4,258	11,905

**Table S4:** Additional prosthesis costs (undiscounted) of MPKs vs NMPKs.

Time horizon (years)	Germany: C-Leg vs. NMPKs		Sweden: Kenevo vs. NMPKs	Netherlands: MPKs vs. NMPKs
	With DM	Without DM		
0.5	18,784	18,784	7,670	16,601
1	18,784	18,784	7,670	16,601
2	18,598	18,600	7,580	16,523
3	17,777	17,713	7,294	16,180
4	16,423	16,102	8,941	15,617
5	15,002	14,243	8,806	15,033
6	13,876	12,626	8,692	14,574
7	23,565	28,347	10,369	20,973
8	22,863	27,137	10,305	20,683
9	22,212	25,916	10,255	20,415
10	21,628	24,723	10,621	20,175
11	21,124	23,605	10,591	19,969
12	20,689	22,560	10,567	19,791
13	25,201	35,067	10,977	22,602
14	24,865	34,118	10,962	22,464
15	24,571	33,217	10,950	22,344
16	24,316	32,367	11,050	22,239
17	24,093	31,566	11,043	22,147
18	23,898	30,810	11,036	22,068
19	26,009	40,643	11,159	23,312
20	25,860	39,971	11,155	23,251
21	25,731	39,339	11,152	23,198
22	25,618	38,745	11,185	23,151
23	25,520	38,187	11,182	23,111
24	25,434	37,665	11,180	23,076
25	26,411	45,184	11,224	23,621

## AUTHORS BIOGRAPHY

**Prof. Dr. Alexander Kuhlmann**

Alexander Kuhlmann is Professor of Health Economics and head of the health economic research unit at the University of Lübeck. After studying economics and completing his PhD, he was postdoc at Leibniz University Hannover. From 2021-2024, he was Assistant Professor of Health Economics at Martin Luther University Halle-Wittenberg, leading the research groups Health Economic Evaluation and Mathematical Modelling in Medical Sciences. Alexander Kuhlmann has long-standing experience and expertise in health economic modelling of medical interventions / public health programs in infectious and non-infectious diseases. For his health economic evaluation of the Kenevo microprocessor-controlled prosthetic knee, he was awarded the Forchheimer Prize.

**Dr. Andreas Kannenberg, MD (GER), PhD**

Andreas Kannenberg graduated from Charité Medical School at Humboldt University in Berlin and worked as a physician in Germany for more than 10 years. He joined Ottobock in 2003 and has been serving as Executive Medical Director North America since 2013. He coordinates Ottobock's clinical research in the Americas and provides medical support to negotiations with health care payers in the US and Canada. He was presented AOPA's Thranhardt Award in 2010, 2023, and 2024, AOPA's Hamontree Award in 2023, and awarded the Honorary Membership of the American Academy of Orthotists & Prosthetists (AAOP) in 2024.
